# Determination of Chitin Content in Insects: An Alternate Method Based on Calcofluor Staining

**DOI:** 10.3389/fphys.2020.00117

**Published:** 2020-02-18

**Authors:** Bianca Santos Henriques, Eloi Souza Garcia, Patricia Azambuja, Fernando Ariel Genta

**Affiliations:** ^1^Laboratory of Insect Physiology and Biochemistry, Oswaldo Cruz Institute – Oswaldo Cruz Foundation (IOC-FIOCRUZ), Rio de Janeiro, Brazil; ^2^National Institute of Science and Technology for Molecular Entomology (INCT-EM), Cidade Universitária, Rio de Janeiro, Brazil

**Keywords:** chitin, polysaccharide, calcofluor, insect, quantitation, technique

## Abstract

Chitin is an aminopolysaccharide present in yeast cells and arthropod cuticle and is one of the most abundant biopolymers. The conventional methods for the quantitation of chitin content in biological samples are based on its hydrolysis (acid or enzymatic), and the assessment of the byproduct, glucosamine. However, previously described methodologies are time-consuming, laborious, low throughput, and not applicable to insect samples in many cases. Here we describe a new approach to chitin content quantitation based on calcofluor fluorescent brightener staining of samples, followed by microplate fluorescence readings. Calcofluor is a specific chitin stain commonly used for topological localization of the polymer. The protocol was tested in three important disease vector species, namely *Lutzomyia longipalpis*, *Aedes aegypti*, and *Rhodnius prolixus*, and then compared to a classic colorimetric chitin assessment method. Results show that chitin content in the tested insects can vary largely in a range of 8–4600 micrograms of chitin per insect, depending on species, sex, and instar. Comparisons between measurements from the previous protocol and calcofluor method showed statistically significant differences in some samples. However, the difference might be due to interference in the classic method from non-chitin sources of glucosamine and reducing agents. Furthermore, chitinase hydrolysis reduces the total chitin mass estimated between 36 and 74%, consolidating the fluorescent measurements as actual stained chitin in the same extent that was observed with the standard protocol. Therefore, the calcofluor staining method revealed to be a fast and reliable technique for chitin quantitation in homogenized insect samples.

## Introduction

Chitin is a large hydrophobic homopolymer chain of β-(1-4)-linked *N*-acetyl-D-glucosamine synthetized by membrane-bound chitin synthase enzymes (EC 2.4.1.16) (Cohen, 2010). It is an aminopolysaccharide present in both fungal cell wall and arthropod exoskeleton, such as insects, and is responsible for providing rigidity and structural integrity (Cohen, 1993, 2010; Pillai et al., 2009).

The determination of chitin amounts has been previously described mainly for yeast cells. However, it has been done based on the assessment of respective glucosamine release through chitin acid hydrolysis (Paech and Tracey, 1956; Chen and Johnson, 1983) or deacetylation (Ride and Drysdale, 1972; Lehmann and White, 1975), which are laborious and time-consuming processes.

Chitin quantitation was also achieved through the enzymatic hydrolysis of chitin and chitosan, still configuring a demanding and challenging technique (Subramanyam and Rao, 1987). Other methods propose a direct quantification of chitin levels through flow cytometry in fungi cells stained with specific chitin dye, calcofluor white (Costa-de-Oliveira et al., 2013). Nevertheless, this approach is not applicable to insect samples.

Calcofluor white is a fluorescent brightener widely used in the topological localization of chitin, commonly used in yeast fluorescent microscopy. This fluorochrome has the ability to make hydrogen bonds oriented to the structural polysaccharide microfibrils (Herth and Schnepf, 1980; Herth, 1980).

Here we describe a fast and simple protocol to determine chitin content in homogenized insect samples stained with calcofluor, based on fluorometric microplate readings.

## Materials and Equipment

### Materials

Steel beads (11079112ss, BioSpec Products); calcofluor white fluorescent brightener (F3543, SIGMA); *Streptomyces griseus chitinase* (C6137, Sigma).

### Equipment

Mini-Bead Beater (BioSpec Products); fluorimeter microplate reader (SpectraMax^®^ Gemini XPS, Molecular Devices).

## Materials and Methods

### Rhodnius prolixus

*Rhodnius prolixus* were reared in the Laboratory of Insect Biochemistry and Physiology (LABFISI – IOC/FIOCRUZ) maintained at 28°C and relative humidity between 60 and 70%. Insects were fed through artificial apparatus upon latex membrane with defibrinated rabbit blood (Garcia et al., 1984). Blood was provided by the Laboratory Animals Creation Center of Fiocruz (Cecal), respecting the guidelines established by the Ethics Committee on Animal Use (CEUA) established by Fiocruz (license number LW019/17). Samples were prepared using starving first instar nymphs collected 7 days after egg hatching, or starving male adults (at least 2 weeks from the last blood meal).

### Lutzomyia longipalpis

Adult sandflies were obtained from a colony descendant from individuals initially captured in Jacobina, Brazil, maintained at LABFISI. Larvae were fed with a mix of rabbit feces, rabbit food, and soil throughout all larval stages, with the addition of white soy protein (bran) and cereal flakes (Neston) (1:1) during third and fourth instars. After molting, adults were kept with 70% sucrose solution (w/v), and females were blood fed in anesthetized hamster (Ketamine, 200 mg/kg) (CEUA L-029/2016) (Moraes et al., 2014). Samples were prepared with adult males or females collected 3–5 days after emergence from pupae, and fed with sugar only.

### Aedes aegypti

Eggs from the Rockefeller strain were obtained from the Laboratory of Physiology and Control for Arthropod Vectors (LAFICAVE – IOC/FIOCRUZ, License CEUA L-004/2018). The hatching of eggs was induced by the addition of distilled water in plastic cups. Larvae were reared until the adult stage in dechlorinated water containing 0.1 g of cat food (Whiskas^®^, Purina, Brazil) in LABFISI at 28°C and relative humidity between 60 and 70% with 12-h light and dark cycle (Souza et al., 2016). Samples were prepared with starving adult males or females collected 1–2 days after emergence.

### Chitin Measurement

#### Preparation of Samples

Insects were homogenized using a Mini-Bead Beater (BioSpec Products) and 1.2 mm stainless steel beads (11079112ss, BioSpec Products). *R. prolixus* first instar nymphs were pooled in groups of 10 in 200 μL, and male adults were homogenized individually in 1000 μL distilled water, followed by samples dilution. *L. longipalpis* were grouped in pools of 20 insects in 200 μL distilled water and *A. aegypti* adults in pools of 20 insects in 200 μL distilled water.

#### Lehmann and White Method (1975)

Chitin content from samples was determined according to Lehmann and White (1975). Aliquots of 20 μL of *R. prolixus* samples, or 40 μL for *L. longipalpis* and *A. aegypti*, were centrifuged at 21,000 × *g* for 5 min at room temperature. Pellets were resuspended in 200 μL 3% (w/v) sodium dodecyl sulfate and incubated at 100°C for 15 min. After cooling, samples were centrifuged again, washed twice with 200 μL water and resuspended in 150 μL of 2.1 M KOH. Samples were heated to 130°C for 1 h then brought to room temperature for the addition of 400 μL 75% (v/v) ethanol and incubation on ice for 15 min. After that, 60 μL of the supernatant of 1 g suspension of Celite^®^ 545 in 12.5 mL 75% ethanol (rested for 2 min) were added to the samples, following centrifugation and washing with 400 μL 40% ethanol solution.

Pellets containing insoluble chitosan were then resuspended in 125 μL water, plus 125 μL of 5% (w/v) NaNO_2_ and 125 μL 5% (w/v) KHSO_4_ and samples were incubated at room temperature for 15 min. The same was done with 125 μL water as a control. After centrifugation (1,500 × *g*, 2 min, 4°C), two aliquots were taken from each tube as replicates and combined each with 50 μL of ammonium sulfamate 12.5% (w/v) and 50 μL of fresh 12.5% (w/v) MBTH (3-methyl-benzo-2-thiazolone-hydrazone). Samples were incubated at 100°C for 3 min and left to cool, then 50 μL of 0.83% (w/v) FeCl_3_⋅6H_2_O, and samples were incubated at room temperature for 25 min. Aliquots of 200 μL were transferred to a 96-well microplate and absorbance at 650 nm was measured using a spectrophotometer (SpectraMax^®^ 190, Molecular Devices). When necessary, samples were diluted in water to reduce optical density to the range of the reader.

#### Calcofluor Method

Aliquots were taken from the different species samples and added to 100 μL of a solution of calcofluor white fluorescent brightener (F3543, SIGMA), at a concentration of 1 mg/mL for *R. prolixus* nymphs and *L. longipalpis* adults or 50 mg/mL in dimethyl sulfoxide (DMSO) for *R. prolixus* adult males and *A. aegypti* adults. Higher concentration of calcofluor was used for the last samples due to an observed underestimation of the amounts of chitin (see section “Discussion”). Experiments required different volume input for each type of sample (Table 1).

**TABLE 1:** Detailed methodology for the three insect species, *Rhodnius prolixus*, *Lutzomyia longipalpis*, and *Aedes aegypti*.

					**Volume input**		**Insect equivalent input**	
						
**Insect Species**	**Stage**	**Sex**	**Number of insects**	**Total H_2_O volume (μL)**	**Lehmann and White (μL)**	**Calcofluor quantitation method (μL)**	**Lehmann and White**	**Calcofluor quantitation method**
*R. prolixus*	First instar	-	10	200	80	20	0.8	0.2
	*adult*	Male	1	1000	20	15	0.004	0.003
*L longipalpis*	Adult	Male	40		50	25		
				200			2	1
		Female	20		100	50		
*A. aegypti*	Adult	Male			50	50	1	1
			20	200				
		Female			20	30	0.4	0.6

*Data includes stage, sex, number of insects, total volume of distilled water for sample homogenization, volume input in quantitation assays after chitinase hydrolysis incubation and number of insects contained in volume input for chitin determination (considering sample dilution).*

Samples were incubated in the dark for 15 min for chitin binding to the stain, then centrifuged 21,000 × *g* for 5 min. and the pellets were washed two times with 200 μL distilled water. Pellets were resuspended in 200 μL distilled water and transferred to a black 96-well microplate (Costar, 3915, Corning). Fluorescent intensity was determined using a microplate reader (SpectraMax^®^ Gemini XPS, Molecular Devices) with excitation at λex 355 nm and emission at λem 433 nm.

##### Chitin hydrolysis

Samples of *R. prolixus* first instar nymphs and adult males; adult males and females of *L. longipalpis;* and adult males and females of *A. aegypti* were prepared (see item “Preparation of Samples” for details). Aliquots of 70 μL were separated and combined with 70 μL of 1 mg/mL chitinase from *Streptomyces griseus* (C6137, Sigma) in 50 mM potassium phosphate, pH 6.0, 20 mM sodium azide. Control groups consisted of tubes containing samples and 70 μL of the same buffer. Samples were incubated at 37°C for 18 h, then filled to 350 μL with distilled water and aliquots were taken for chitin content determination following both protocols.

### Preparation of Colloidal Chitin Suspension

Colloidal chitin suspension was prepared following previously described methodology (Hsu and Lockwood, 1975). Briefly, 2 g of commercial chitin from shrimp shells (C7170, SIGMA) were dissolved in 40 mL of concentrated hydrochloric acid (36.5–38%, Vetec, V000154) by magnetic stirring for 50 min. Chitin was then precipitated by adding it to 100 mL of distilled water. The colloidal suspension was centrifuged, and the pellet washed with distilled water until pH was above 3.5.

### Determination of Colloidal Chitin Suspension Concentration

For the determination of the colloidal chitin suspension concentration, masses between 1 and 20 mg of chitin (C7170, SIGMA) and 400 μL of distilled water were added to glass test tubes, followed by the addition of 400 μL of phenol 5% (w/v). Then one milliliter of sulfuric acid (95–97%) was pipetted rapidly directly onto the liquid surface, in order to obtain proper mixing of the reagents. Tubes were incubated for 10 min, shaken and incubated for 10 more min at 30°C (Dubois et al., 1956). Volumes of 200 μL of samples were transferred to a 96-well microplate and absorbance at 490 nm was measured using a spectrophotometer (SpectraMax^®^ 190, Molecular Devices). Chitin weight was plotted against corresponding absorbance values resulting in a standard curve. The same was done using samples of colloidal chitin to confirm concentrations. Slopes from the two curves were used to estimate the suspension concentration.

### Standard Curve and Chitin Content Determination

For the calculations of the amount of chitin recovered from samples, a standard curve was prepared using increasing volumes of colloidal chitin suspension at 4.24 μg/μL, by plotting the weight of chitin added against corresponding absorbance or fluorescence. Chitin content was determined using linear regression prediction, taking into account dilution, sample volume input and volume per insect.

### Statistical Analyses

All data obtained from chitin quantitation assays were analyzed by Two-way analysis of variance (ANOVA) by using the software GraphPad Prism version 6.04 for Windows (San Diego, CA, United States). All quantitation graphs denote means of at least ten, up to 40 samples, and respective standard errors of the means (SEM).

## Results

**FIGURE 1:**
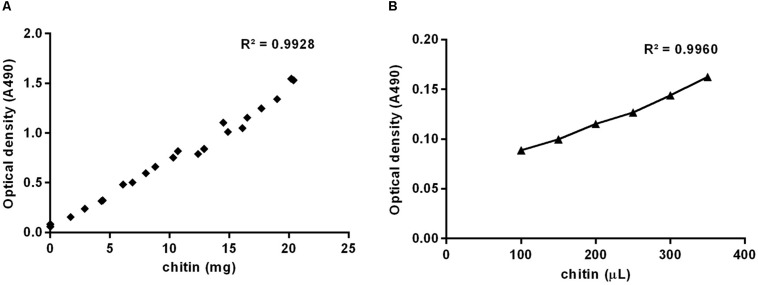
Standard curves prepared using increasing masses of powdered chitin from shrimp shells **(A)** or increasing volumes of colloidal chitin suspension **(B)**, based on optical density at 490 nm.

A colloidal chitin suspension was prepared according to Hsu and Lockwood (1975) to be used as a standard for the estimation of the unknown chitin content from samples. Authors infer that the protocol for the suspension preparation recovers at least 85% of the chitin input dissolved in Hydrochloric acid, therefore, the concentration of the suspension needed to be evaluated. We used the colorimetric method for the determination of sugars described by Dubois et al. (1956), applying the protocol to increasing masses of powdered chitin from shrimp shells (Figure 1A) or increasing volumes of the colloidal chitin suspension prepared (Figure 1B). Weights and volumes, respectively, were plotted against optical density obtained at 490 nm, and slopes used to estimate the concentration of the suspension at 4.24 μg/μL.

**FIGURE 2:**
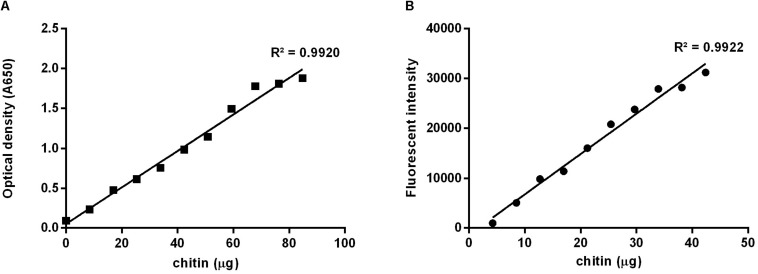
Standard curves prepared using increasing masses of colloidal chitin suspension at 4.24 μg/μL. **(A)** Based on the method described by Lehmann and White (1975), absorbance at 650 nm; **(B)** Using calcofluor stained chitin, excitation at λex 355 nm and emission at λem 433 nm.

For the calculation of the corresponding amount of chitin contained in the tested samples, it was necessary to build standard curves (Figure 2) applying both methods to increasing masses of chitin suspended in a colloidal suspension. Weights of chitin were plotted against the optic density, for the Lehmann and White (1975) methodology (Figure 2A), or against the fluorescent intensity (Figure 2B), for the calcofluor staining quantitation method. Colloidal chitin suspension contained 4.24 μg/μL, and curves presented slopes of 0.02 and 809.16, respectively, both with *R*^2^ = 0.99.

Linear regression was applied to estimate the unknown amount of chitin in the tested samples by fitting the obtained optical density or fluorescence to the respective standard curves. Results were then subjected to adjustments considering the number of insects homogenized, the total volume used, the volume input in the chitinase hydrolysis step, and volume input in each quantitation assay.

For the purpose of testing the application of the described method in homogenized insect samples, we used three insect species, that are important disease vectors and research models, namely *Lutzomyia longipalpis*, *Aedes aegypti*, and *Rhodnius prolixus*. Aliquots of homogenized samples were submitted to hydrolysis using a commercial chitinase enzyme from the bacterial species *Streptomyces griseus.* This step was included in order to verify if the enzyme activity would reduce the total amount of chitin calculated using both methods, and make sure what was being measured was, in fact, the desired polysaccharide.

**FIGURE 3:**
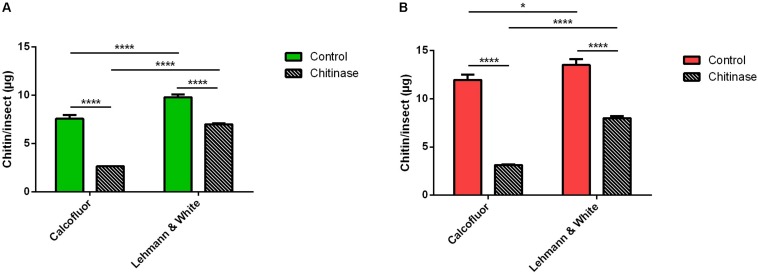
Chitin content assessment in adult *Lutzomyia longipalpis* pooled in groups of 20–40 in 200 μL distilled water, treated or not with 1 mg/mL chitinase from *Streptomyces griseus*, comparing measurements with the methods by Lehmann and White (1975) and calcofluor fluorescent brightener. **(A)** Male *L. longipalpis*. **(B)** Female *L. longipalpis*. Bars are means of 20 biological replicates with the standard error of the mean (SEM). Asterisks denote statistically significant differences, *p ≤ 0.05, and ****p ≤ 0.0001.

The assessment of the chitin content of samples containing a pool of 40 males or 20 females of *L. longipalpis*, homogenized in 200 μL of distilled water, resulted in similar amounts of chitin in both methodologies (Figure 3). Quantitation assays were carried out in both control samples and samples incubated in the presence of chitinase. Using the calcofluor staining method we obtained a result of 7.57 ± 0.38 micrograms of chitin per insect for control males and 2.64 ± 0.02 μg/insect for the chitinase hydrolyzed samples, a reduction of 65% in the total mass of the polysaccharide (Δ = 4.93 μg/insect) (Figure 3A). Meanwhile, using the Lehmann and White method in the same group of insect the resulting measurements were 9.79 ± 0.30 μg/insect for control and 6.99 ± 0.12 μg/insect for the chitinase treated group (Figure 3A), meaning a reduction of 29% (Δ = 2.80 μg/insect).

In the female *L. longipalpis* control group, the quantitation with the fluorescent stain resulted in a total mass of 11.96 ± 0.54 μg of chitin/insect, while the chitinase treated group resulted in 3.13 ± 0.02 μg/insect (Figure 3B), accounting for a reduction of 74% (Δ = 8.83 μg/insect). For the Lehmann and White chitin assessment method, in the control group results showed a total of 13.50 ± 0.61 μg/insect, and 7.99 ± 0.22 μg of chitin per insect after hydrolysis (Figure 3B), reducing the polymer content calculated in this group of insects by 41% (Δ = 5.51 μg/insect).

**FIGURE 4:**
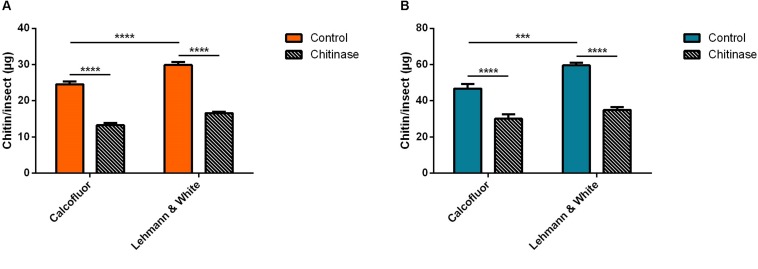
Chitin content assessment in adult *Aedes aegypti* pooled in groups of 20 in 200 μL distilled water, before and after treatment with 1 mg/mL chitinase from *Streptomyces griseus*. We also compare measurement methods by Lehmann and White (1975) and the calcofluor fluorescent brightener. **(A)** Male *A. aegypti*. **(B)** Female *A. aegypti*. Bars are means of 30 biological replicates with the standard error of the mean (SEM). Asterisks denote statistically significant differences, ***p ≤ 0.001, and ****p ≤ 0.0001.

For *Aedes aegypti*, adult males or females were gathered in pools of 20 insects and subsequently homogenized in a total volume of 200 μL of distilled water. Samples were divided into aliquots for the control groups and treatment with chitinase (Figure 4). Using calcofluor fluorescent brightener for the total chitin mass assessment in control samples of male *A. aegypti* resulted in 24.6 ± 0.7 μg of chitin/insect (Figure 4A). Chitinase treatment reduced the polysaccharide amount by 46%, resulting in 13.2 ± 0.6 μg/insect (Δ = 11.3 μg/insect) (Figure 4A). The method described by Lehmann and White (1975) for the male *A. aegypti* control group resulted in a total of 29.9 ± 0.8 μg of chitin per insect, while the chitinase treatment reduced it by 44%, with a total residue of 16.6 ± 0.4 μg/insect.

Female *A. aegypti* chitin measured with calcofluor gave a mean of 47 ± 3 μg/insect (Figure 4B). Treatment with chitinase from *S. griseus* reduced the polymer detection by 36%, corresponding to 30 μg of chitin/insect (Figure 4B). Colorimetric quantitation by deacetylation (Lehmann and White, 1975) resulted in a calculated mass of 60 ± 2 μg of chitin/insect in the control group, while the chitinase treated group had a value corresponding to 35 ± 2 μg/insect after treatment (Figure 4B). In this case, hydrolyzation accounted for a reduction of 41% in the estimated total mass of chitin (Δ = 25 μg/insect).

**FIGURE 5:**
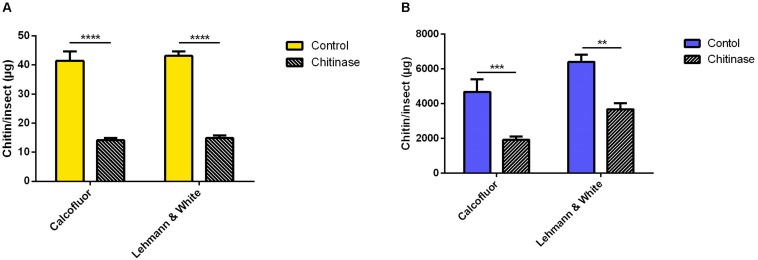
Chitin content assessment in *Rhodnius prolixus* treated with 1 mg/mL chitinase from *Streptomyces griseus*, comparing measurement methods by Lehmann and White (1975) and calcofluor fluorescent brightener. **(A)** First instar nymphs pooled in groups of 10 in 200 μL distilled water **(B)** Adults homogenized individually in 1 mL distilled water. Bars are means of 20 or 10 biological replicates for nymphs or adults, respectively, with the standard error of the mean (SEM). Asterisks denote statistically significant differences, **p ≤ 0.01, ***p ≤ 0.001, and ****p ≤ 0.0001.

In the case of the hemipteran species *R. prolixus*, chitin quantitation in first instar nymphs was performed in homogenized pools of 10 insects in 200 μL of distilled water. After incubation with chitinase, sample’s chitin content was assessed comparatively using both fluorometric and colorimetric assays (Figure 5). Calcofluor staining resulted in an estimated total mass of chitin of 41.4 ± 3.3 μg/insect in the control group, and 14.1 ± 0.8 μg of chitin per insect after chitinase treatment (Figure 5A). Hydrolysis resulted in a reduction of 66% (Δ = 27.3 μg/insect). Comparatively, the previously described quantitation method estimated a total of 43.1 ± 1.5 μg/insect for control samples, and 14.9 μg/insect after chitinase hydrolyzation (Figure 5A), equally amounting to a 66% reduction in chitin concentration (Δ = 28.3 μg/insect).

For adults of the same species, males were homogenized individually in 1 mL of distilled water and processed similarly to previous groups. Chitin mass assessment using the calcofluor method estimated a mean of 4600 ± 700 μg/insect, and 1900 ± 100 μg of chitin/insect after sample hydrolysis with chitinase (Figure 5B). Using the Lehmann and White (1975) method, the quantitation of chitin in *R. prolixus* adult males totalized a mass of 6406 ± 416 μg/insect in the control group, and 3682 ± 350 μg/insect following the chitinase incubation step (Figure 5B). Polysaccharide hydrolysis resulted in a reduction of 59% (Δ = 2753 μg/insect), and 43% (Δ = 2724 μg/insect), respectively.

Alternatively, similar results were obtained by homogenizing bigger insects using a mortar and pestle. Tissues were covered with liquid nitrogen, homogenized while frozen and resuspended in distilled water. For smaller insects, homogenization using a pellet pestle cordless motor (Z359971 Sigma) also gave similar results (data not shown). All measurements are summarized in Table 2.

**TABLE 2:** Chitin amounts per insect in three different species, as measured by with Lehmann and White and Calcofluor white staining techniques.

			**Lehmann and White microplate procedure**			**Calcofluor white staining**		
				
			**Before chitinase**	**After chitinase**		**Before chitinase**	**After chitinase**	
			**treatment (μg)**	**treatment (μg)**		**treatment (μg)**	**treatment (μg)**	
**Insect Species**			**mean ± SEM**	**mean ± SEM**	**Net change**	**mean ± SEM**	**mean ± SEM**	**Net change**
*R. prolixus*	First instar	– Male	43 ± 2	14.9 ± 0.9	28.3	41 ± 3	14.1 ± 0,8	27.3
	Adult		6406 ± 416	3682 ± 350	2724	4669 ± 729	1916 ± 198	2753
*L. longipalpis*	Adult	Male	9.8 ± 0.3	7.0 ± 0.1	2.80	7.6 ± 0.4	2.64 ± 0.02	4.93
		Female	13.5 ± 0.6	8.0 ± 0.2	5.51	12.0 ± 0.5	3.13 ± 0.07	8.83
*A. aegypti*	Adult	Male	29.9 ± 0.8	16.6 ± 0.4	13.2	24.6 ± 0.7	13.2 ± 0.6	11.3
		Female	60 ± 2	35 ± 2	25	47 ± 3	30 ± 2	17

*Measurements were performed in crude homogenates, before and after treatment with commercial chitinase from *Streptomyces griseus*. Values are means with standard error of the mean (SEM), μg per insect, from at least 10 biological replicates. See section “Materials and Methods” for details.*

## Discussion

Chitin is a polysaccharide composed of an extended hydrogen-bonded chain of repeated β-(1-4)-linked *N*-acetyl-D-glucosamine. This biopolymer is one of the most abundant regarding biomass in nature, second only to cellulose, being synthesized by numerous living organisms, mainly arthropods, and fungi (Rinaudo, 2006; Cohen, 2010). Amongst the Arthropoda, insects rely on this bioproduct for fundamental physiological functions, and it is present in various extracellular structures, such as the integument (Cohen, 2010).

Insect cuticle is composed of layers of chitin separated by layers of protein, as X-ray analyses data suggested (Fraenkel and Rudall, 1946), possibly containing variations of the purely poly-*N*-acetyl-glucosamine, with about one glucosamine in six residues, and about 5% bound water even in thoroughly dried samples (Rudall, 1963). Generally, protein is the predominant compound in insect cuticle, corresponding to a range of 40–75% (p/p) of crude protein on dry weight basis, varying depending on the species and life cycle stage (Finke, 2007). In the exuviae, protein varies between 44 and 61%, and chitin can account for a range of 30–42% (Kramer et al., 1995).

This biopolymer has become a new and attractive target for pest control, because of its differential distribution amongst living organisms, which would reduce possible unwanted health risks for vertebrates (Cohen, 1993). The insecticide class of Insect Growth Regulators was introduced in 1972 with the discovery of the effects of Diflubenzuron, the first prototype for the benzoylurea chitin synthesis inhibitor insecticides (Verloop and Ferrell, 1977). Because of the potent insecticidal activities of the class, chitin quantitation emerges as an important diagnosis of the effects of these compounds in insect chitin physiology. Besides that, a rapid and fast chitin measurement technique would allow the high throughput exploration of resistance mechanisms linked to changes in chitin quantity, composition and structure in field samples. It is noteworthy to point out that resistance against chitin synthesis inhibitors was already stablished in mosquitoes (Araújo et al., 2019; Porretta et al., 2019) and other insects (Wang et al., 2019), and that changes in cuticle composition are the basis of an important resistance mechanism related to reduced penetration of toxic molecules (Balabanidou et al., 2018).

Chitin quantitation has been proposed through different approaches, mainly focusing on the application of the methods in fungal cell wall analyses. Protocols described chitin acid hydrolysis using hydrochloric acid (Paech and Tracey, 1956; Chen and Johnson, 1983) or deacetylation through high-temperature incubation with saturated potassium hydroxide (Ride and Drysdale, 1972; Lehmann and White, 1975) and measured the chitin content colorimetrically from samples as the equivalent in glucosamine released, using glucosamine standard curves. Alternative methods emerged, proposing the direct assessment of the polysaccharide in question using the products of chitinase hydrolyzates (Subramanyam and Rao, 1987). However, still correlating the total amount of chitin to the *N*-acetyl-glucosamine released by the hydrolysis catalyzed by the enzyme. Moreover, despite being applicable to insect samples, these protocols are time-consuming, extremely laborious, and low-throughput, which reduces the number of samples that can be processed at the same time.

Direct chitin content evaluation has also been achieved in an analytical chemistry approach, such as the use of micro distillation (Kan et al., 1964), gas chromatography (Sweeley et al., 1963), or colloidal titration (Tôei and Kohara, 1976). Alternatively, previous works involving the use of insects as sustainable nutritional sources calculate the chitin concentration as the corrected measurement for the acid detergent fiber (ADF) fraction minus the percentage that corresponds to amino acids (Finke, 2002). This method of analysis can overestimate the quantitation, because the amino acids methionine, cysteine, and tryptophan were not included in the ADF fraction measurement (Finke, 2007).

The use of the chitin specific stain, calcofluor fluorescent brightener, has been previously proposed while associated with the use of flow cytometry to quantify the amount of chitin present in the fungal cell wall (Costa-de-Oliveira et al., 2013). Authors quantified the fluorescent intensity of stained cells in some yeast strain isolates, and results are showed as a mean staining index. Furthermore, Costa-de-Oliveira et al. (2013) infer that measurements are in agreement with previous works (Bulawa et al., 1995; Plaine et al., 2008; Walker et al., 2008), but the arbitrary units used in the data seem to be difficult to extrapolate.

With the known use of calcofluor for topological localization of chitin in fungal cells and the known affinity of the stain to its target, we were able to quantify successively small amounts of chitin in homogenized samples of the proposed insect species. Our calcofluor staining method results in calculated measurements for insects individually, through microplate fluorescent intensity readings. An adapted protocol of Lehmann and White (1975) for microplate assays was chosen as a comparative control method. Several previous works involving chitin assessment in insect samples have used the Lehmann and White technique (Lehmann and White, 1975; Zhang and Zhu, 2006; Rezende et al., 2008; Farnesi et al., 2012; Chen et al., 2013), and it has been similarly applied to other arthropods (Hackman and Goldberg, 1981). However, to make the measurements obtained with both techniques comparable to each other, we decided to use colloidal chitin instead of glucosamine for the calibration curves.

Our results show that *L. longipalpis* females have more chitin content than males, with a composition of about 12 μg of chitin per insect, while the individual male body is composed of about 8 μg of chitin. The same tendency is seen in *A. aegypti*, with females having about 47 μg chitin/insect, and males around 25 μg of chitin per insect. The females tend to be larger than males in both cases and may have eggs in the ovaries (Farnesi et al., 2015; Souza et al., 2019), which would account for the differences seen in measurements. *Rhodnius prolixus* first instar nymphs have 41 μg of chitin/insect, and adult males increase this total to a mean of 4600 μg/insect, an enhancement of 1000 fold in the concentration of the polymer, which is consistent with the growth of the insects.

For *L. longipalpis* and *A. aegypti* females the measured chitin content corresponds to about 3 and 4% of the insects’ total wet weight, respectively, and for *R. prolixus*, it corresponds to 10% for first instar nymphs’ wet weight and 8% for adult males. The percentage of the insect weight that corresponds to chitin is similar to what is seen in other insect species. Previous works described that amongst the studied species the chitin content could range from 0,27%, as is the case of the bee brood, to 5% in mealworm beetles (Finke, 2007, 2013).

Comparisons between the two methods resulted in similar amounts of chitin in all tested cases, although some statistically significant differences were observed. Differences may be due to interference in the control method caused by the presence of glucosamine from non-chitin sources. Subramanyam and Rao (1987) verified that a significant part of the measured chitin accounts for hexosamine contaminants, even in commercialized chitin.

Chitinase hydrolysis was used as a proof of concept, showing that the fluorescence obtained from calcofluor stained samples related to the amount of chitin present. As expected, in all species, incubation with the enzyme resulted in a significant reduction of the total amount of chitin estimated by the calculations. Residual amounts of staining were observed in all hydrolyzate samples, possibly because of the unbalanced ratio of enzyme to the substrate (≥0.0002 units per microliter of the sample) or the incubation length (18 h). Chitinase preparations from cockroaches may take up to 6 days to digest about 10% of the substrate (Waterhouse et al., 1961). Nevertheless, when saturated to ten units of enzyme per nmol of chitin, chitinase is able to completely solubilize the chitin present in fungal cell walls in 10 h (Subramanyam and Rao, 1987). In *L. longipalpis*, the signal reduction after hydrolysis showed to be less effective in the colorimetric method when compared to calcofluor staining. This data probably suggests that the chitinase activity in this particular sample results in the noteworthy production of free glucosamine, which has been recorded to represent up to about 10% of chitin digests (Waterhouse et al., 1961). Besides that, the treatment with chitinase seems to be less effective in *A. aegypti* samples with respect to the other species analyzed (Table 2).

Interestingly, the calcofluor fluorescent brightener at a concentration of 1 mg/mL was efficient in the quantitation of smaller insects, namely both males and females *L. longipalpis*, and *R. prolixus* first instar nymphs. However, in *R. prolixus* adults, and *A. aegypti* males and females, we observed underestimations in chitin quantitation (data not shown). This may be related to the higher amount of chitin present in the insect’s body (*R. prolixus*) or to different accessibility of the chitin microfibrils to the stain (*A. aegypti*). Dilution of the samples resulted in low reproducibility, probably because of the difficulty of getting homogeneous aliquots from a diluted suspension. Therefore, the stain concentration was enhanced to 50 mg/mL, and for that, the solvent of the stock solution was changed to DMSO. In this way, the sample and calcofluor concentration need to be taken into account, since chitin saturation may result in a low polymer/stain ratio, and, consequently, ineffective quantitation due to underestimation. Thus the volume input was represented as an insect equivalent (Table 1), meaning the number of insects per sample that are necessary for a correct quantitation.

Doubling sample concentrations returns doubled fluorescent intensity signal, but does not change final estimation in chitin concentration per insect (data not shown). It is important to notice that the high background observed after chitinase treatment was similar in both techniques tested, meaning that calcofluor might be used for relative comparison between insects groups in the same way as the Lehmann and White technique. Interestingly, the net change observed after chitinase treatment was very similar for both techniques (Table 2), suggesting that they are detecting the same chemical entity. In the future, it would be important to characterize the source of this residual background, as it would correspond to undigested chitin or other glucosamine containing molecules, as glycoproteins or glycolipids.

The chitin determination protocol described herein constitute a simple, fast, and considerably less laborious method in comparison with previous quantitation methods. However, the necessity of a more sophisticated equipment, as a fluorescence microplate reader, may be a restriction for its application in basic-equipped laboratories. Furthermore, analyses demonstrate that the assay is consistent and reliable, suggesting a possible incorporation in screening for insecticide resistance mechanisms against insect growth regulators (IGRs).

## Data Availability Statement

All datasets generated for this study are included in the article/supplementary material.

## Author Contributions

FG and BH conceived and designed the study. BH performed the experiments and data analysis. PA and EG gave important intellectual contributions.

## Conflict of Interest

The authors declare that the research was conducted in the absence of any commercial or financial relationships that could be construed as a potential conflict of interest.
